# 6,7-Dimethoxycoumarin Influences the Erythroid Differentiation of Human Chronic Myelogenous Leukemia K562 Cells through Regulating FOXO3/p27 Signal Pathway

**DOI:** 10.1155/2022/1138851

**Published:** 2022-05-14

**Authors:** Li Zheng, Yu Wu, Fu Wang, Hui Shi, Bo Xu, Aihua Yang

**Affiliations:** ^1^Pharmaceutical Department, Affiliated Maternity and Child Health Care Hospital of Nantong University, Nantong, Jiangsu 226018, China; ^2^Pharmaceutical Department, Nantong Hospital of traditional Chinese Medicine, Nantong, Jiangsu 226001, China; ^3^Traditional Chinese Medicine Department, Nantong Maternal and Child Health Care Hospital, Nantong, Jiangsu 226018, China; ^4^Department of Clinical Pharmacy, Nantong Maternal and Child Health Care Hospital, Nantong, Jiangsu 226018, China

## Abstract

**Objective:**

To study the pharmacological activity and the mechanism of action of natural compounds derived from 6,7-dimethoxycoumarin on the differentiation of human chronic myeloid leukemia K562 cells.

**Methods:**

We use MTT assay (Sigma-Aldrich, USA) to detect cell viability; use flow cytometry to analyze DNA content for cell cycle analysis; use benzidine staining to synthesize hemoglobin to determine K562 cell differentiation; use western blot analysis and qPCR to detect the expression levels of FOX03, P27, CDK4, and their phosphorylation; and use the AOBS laser scanning confocal system (Leica, Wetzlar, Germany) to analyze and quantify the number of positive green spots. The statistical methods used are one-way analysis of variance (ANOVA) and Dunnett's test to analyze within and between groups.

**Results:**

In order to explore the effect of 6,7-dimethoxycoumarin on the differentiation of K562 cell erythrocytes, it was concluded that 6,7-dimethoxycoumarin promotes the differentiation of K562 cell erythrocytes; the proliferation of K562 cells was detected by MTT method, and the results showed that 6,7-dimethoxycoumarin can inhibit the proliferation of K562 cells; to evaluate the effect of 6,7-dimethoxycoumarin on the proliferation of K562 cells, the results showed that 6,7-dimethoxycoumarin increased the expression of FOXO3, P27, CDK4, and CDK65, and decreased the phosphorylation of CDK4 and CDK6 proteins. To further explore the effect of knocking out FOXO3 on cell differentiation, the results show that 6,7-dimethoxycoumarin can reduce the differentiation and proliferation of K562 cells by increasing the expression of FOXO3.

**Conclusion:**

This study extended the understanding of the pharmacological activity of 6,7-dimethoxycoumarin and may provide a potential new target for the treatment of chronic myelogenous leukemia. However, we still need to further study the specific molecular capabilities of 6.7 dimethylcoumarin to understand their possible capture mechanism.

## 1. Introduction

Chronic myelogenous leukemia (CML) is a lethal malignancy. The increased and unregulated growth of predominantly myeloid cells in the bone marrow is the main characteristic of CML [1]. CML has a morbidity of 1-2 cases per 100,000 adults and is responsible for about 15% of all newly diagnosed cases of leukemia in adults. The majority of patients with CML fail to respond effectively to the current regimen of drug therapy due to occurrence of drug resistance [2]. Bone marrow or allogenic stem cell transplantations are recent and effective treatments for CML, but they have a high risk of morbidity and mortality [3]. Tyrosine kinase inhibitor (TKI) is the key to the treatment of chronic myeloid leukemia (CML) at this stage [4]. Compared with previous treatments, it has a higher remission rate and fewer side effects [5]. To a certain extent, the survival rate of patients has been improved. However, the treatment of CML still faces severe challenges. We urgently need new treatment methods to improve the durability of the efficacy and avoid the occurrence of drug resistance.

CML places considerable burden on patients and the majority of chemotherapeutic drugs have long-term side effects [6, 7]. Therefore, it is important to continue research into novel therapeutic approaches for CML. 6,7-Dimethoxycoumarin, also known as Scoparoneis, extracted from a Chinese herb Artemisia scoparia [8]. Coumarin is a natural organic compound. It is mainly derived from the dry aerial parts of Artemisia plants and is often widely used in the treatment of neonatal jaundice [9].

Pharmacological activities of 6,7-dimethoxycoumarin has been broadly studied. Scoparone (6,7-dimethoxycoumarin) is an important chemical substance with a variety of beneficial pharmacological activities, including antitumor, anti-inflammatory, and anticoagulant [10]. It has gained increasing attention for its anticancer activity. It has been demonstrated to be critical in attenuating hepatic injury in Herba Artemisiae Scopariae. In addition, antihypertensive, antianginal effect, and antiatherogenic effect in hyperlipidaemic diabetic has been reported [11–13]. Moreover, 6,7-dimethoxycoumarin is reported to have the property of reducing lipopolysaccharide-mediated TF expression on umbilical vein endothelial cells [14]. However, the role of 6,7-dimethoxycoumarin on erythroid differentiation has not been performed. Some studies have found that scopolone (6,7-dimethoxycoumarin) is purified from the Chinese herbal medicine Artemisia. The results show that it can reduce the proliferation response of peripheral monocytes, relax smooth muscle, and reduce the expression of total cholesterol and triglycerides [15, 16]. It also can delay the pathomorphological changes of diabetic rabbits with characteristic hypercholesterolemia. The ability to remove reactive oxygen species, the inhibition of tyrosine kinases, and the ability to produce prostaglandins have been enhanced [16].

Apoptosis is a conservative cellular suicide. Therefore, the induction of apoptosis is an ideal process as a chemotherapeutic agent and chemopreventive strategy for cancer control. In fact, with the deepening of research on Chinese herbal medicine, more and more researchers are inclined to several plant-derived polyphenol compounds because they may have antioxidant, anticancer, and apoptosis-inducing properties [17–19]. So far, many anticancer drugs used in chemotherapy have developed drug resistance and side effects [19].

Regarding FOXO transcription factors, it includes FOXO1, FOXO3a, FOXO4, and FOXO6 [20]. These related factors in the FOXO family control and participate in various downstream genes that inhibit tumor canceraction [21]. Bim and FasL are used to induce cell apoptosis, and P27 and cyclin D are used to regulate the cell cycle [22]. Activation of FOXO protein is related to cell cycle arrest and induction of cell apoptosis. Therefore, relevant data indicate that FOXO, as a true tumor suppressor gene, may become the most valuable potential therapeutic target for cancer gene therapy. There are literature reports and certain data showing that activating or restoring the activity of FOXO3a and the expression of its downstream genes has become a key target for cancer gene therapy [23].

In this study, we used the commonly used CML chronic granulocyte model K562 cells to evaluate the effect of 6,7-dimethoxycoumarin on red blood cell differentiation. Through a series of confirmatory experiments, we found that 6,7-dimethoxycoumarin promotes the differentiation of red blood cells and effectively inhibits the proliferation of K562 cells. Mechanically, 6,7-dimethoxycoumarin upregulates the expression of FOXO3 and p27, thereby inhibiting the phosphorylation of CDK4/6 and exerting its inhibitory effect on cancer cell proliferation.

## 2. Methods

### 2.1. Cell Culture and Transfection

The human CML cell line K562 were purchased from Chinese Academy of Sciences. Cells were cultured in DMEM medium supplemented with 10% fetal bovine serum (FBS, Hyclone, USA), 4 *μ*m L-glutamine, 100 U/ml penicillin, and 100*μ*g/ml stretomycin. The K562 cells were cultured in a 5% CO_2_ incubator at 37°C. 6,7-Dimethoxycoumarin was purchased from the National Institute for the Control of Pharmaceutical and Biological Products (Beijing, China). The cells were treated with the indicated concentration of 1 *μ*M, 5 *μ*M, and 10 *μ*M 6,7-dimethoxycoumarin and named, respectively, 1 *μ*M, 5 *μ*M, and 10 *μ*M 6,7-Doc group, respectively, and the cells in the control group received an equal volume of DMSO. The final concentration of DMSO was less than 0.1%. siFOXO3 and si-NC plasmids were designed and synthesized by Genepharma (Shanghai, China). Cell transfection was conducted according to the specification of Lipo2000 (Life Technologies, Rockville, MA, USA) for 48-72 hours.

### 2.2. MTT Assay

Cell viability was detected by MTT assay (Sigma-Aldrich, USA). K562 cells were cultured in 96-well plates and incubated with different concentrations of 6,7-dimethoxycoumarin at 1 *μ*M, 5 *μ*M, and 10 *μ*M for 24 h. A total of 10 *μ*l MTT was added to each well and incubated for 3-4 h at a concentration of 5 mg/ml. The MTT solution was removed from the wells by aspiration and the formazan crystals were dissolved in DMSO. A microplate reader was used to measure the optical density at 450 nm, and inhibition rates were calculated as follows: Viability rate (%) = {[A450(sample) − A450(blank)]/[A450(control) − A450(blank)]} × 100. All experiments were performed in triplicate.

### 2.3. Cell Cycle Analysis

Cells were collected after treated with 6,7-dimethoxycoumarin and washed with PBS for 3 times. For the cell apoptosis analysis, cells were stained with 50 mg/L propidium iodide following the manufacturer's instructions. DNA content was analyzed by flow cytometer. The percentage of cell cycle distribution was calculated by Cellquest software (Becton Dickinson, NJ, USA).

### 2.4. Benzidine Staining Assay

K562 cells differentiation was determined by hemoglobin synthesis using a benzidine staining assay. K562 cells were collected followed by washing with cold phosphate buffered saline (PBS) for 2 times. Subsequently, 0.2 ml benzidine solution (2 mg/ml in glacial acetic acid) (Aladdin, Shanghai, China) containing 30% H_2_O_2_ (Aladdin, Shanghai, China) was added to the suspension and further incubated for 10 min at room temperature. The positive cells would be dyed blue which was counted under a microscope (Nikon, Japan).

### 2.5. Western Blot Assay

Total proteins were extracted with RIPA-Buffer (Beyotime Biotechnology, Shanghai, China) supplemented with 10 mM PMSF (Sigma-Aldrich, St Louis, MO, USA) and phosphatase inhibitor (Applygen, Beijing, China). BCA method was carried out to quantify protein concentrations. 40 *μ*g proteins were separated on 10% sodium dodecyl sulfate-polyacrylamide gel electrophoresis (SDS-PAGE) and subsequently transferred to polyvinylidene fluoride (PVDF) membranes. Then, the membrane was blocked with 5% nonfat milk in Tris-buffered saline and 0.1% Tween 20 for 1 h. Subsequently, the membranes were incubated with primary antibodies at 4°C overnight and washed with TBST buffer for 5 times. The membranes were incubated with Horseradish Peroxidase- (HRP-) conjugated secondary antibodies for 2 h. An enhanced chemoluminescence (ECL) system kit (Beyotime, Shanghai, China) was used to visualized using an automatic chemoluminescence image analysis system (Tanon, Shanghai, China). Optical densities (OD) value was analyzed by ImageJ software (NIH, Bethesda, MD, USA).

### 2.6. qPCR Assay

The cell concentration was adjusted to 1 × 10^5^/ml and added 2 ml cell concentration to per well. The cells were collected by trypsinization and centrifugation after 24 h. Total RNA was extracted by TRIzol reagent. cDNA synthesis was referred to Prime script 1st strand cDNA synthesis Kit reverse transcription kit instructions, then cDNA amplification was referred to the instructions for the SYBR Premix Ex Taq TM real-time fluorescent quantitative PCR kit. The primers were designed and synthesized by Shanghai Sangon Biotech Company (Shanghai, China) and listed in Table 1. The 2-△△CT method was used to analyze the expression differences of the target gene among the groups.

### 2.7. Confocal Analysis

K562 cells transfected with siRNA FOXO3 were fixed in 4% paraformaldehyde for 15 min and washed with PBS three times. Cells were then permeabilized with 0.05% (v/v) of Triton X-100 together with DAPI (Invitrogen, Eugene, OR, USA) and analyzed using a AOBS laser scanning confocal system (Leica, Wetzlar, Germany). The number of positive green punctate was quantified.

### 2.8. Statistical Analysis

Results were presented as mean ± standard error (SD) of three independent experiments. Treatment effects were analyzed using Student's *t* test when comparing two variables, One-way analysis of variance (ANOVA) and the Dunnett test were used to analyzed the intra-group and inter-group. Results were considered statistically significant at the probability value of *P* < 0.05 level.

## 3. Results

### 3.1. 6,7-Dimethoxycoumarin Promote the Erythroid Differentiation of K562 Cell

**Figure 1:**
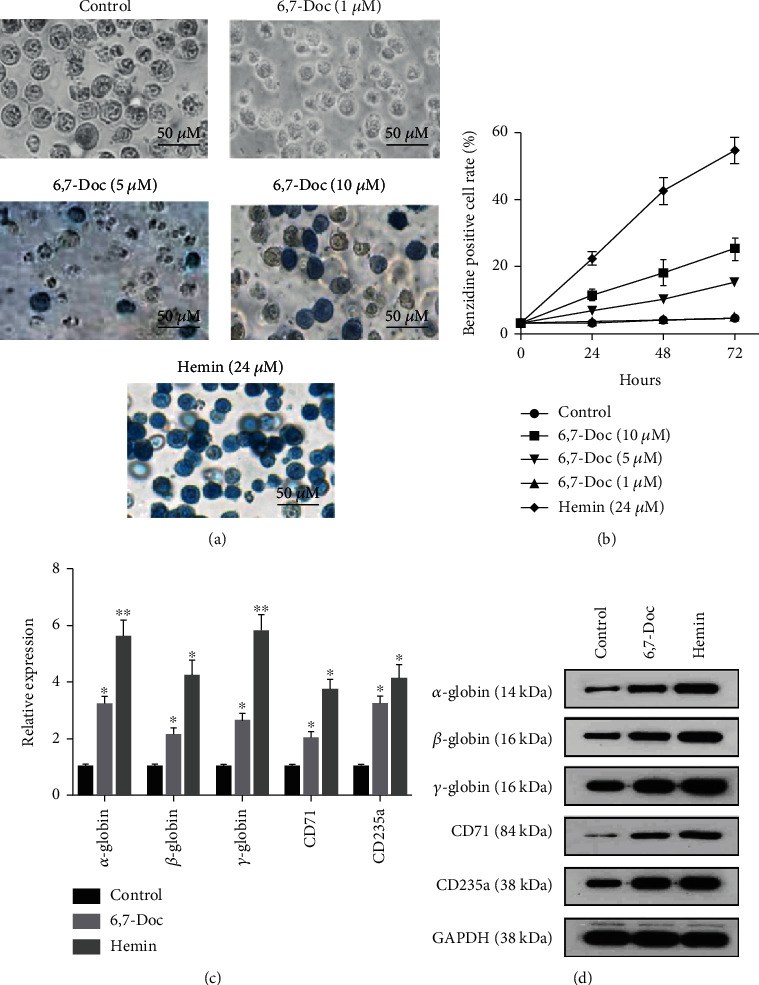
6,7-Dimethoxycoumarin promotes the erythroid differentiation of K562 cell. (a, b) Benzidine staining assay was applied to detect the erythroid differentiation of K562 cells. (c) The expression of typical marker of erythroid differentiation was detected by qPCR assay. (d) The expression of typical marker of erythroid differentiation was detected by western blot assay.^∗^*P* < 0.05, ^∗∗^*P* < 0.01, compared with control group.

To explore the effect of 6,7-dimethoxycoumarin on the erythroid differentiation of K562 cell, benzidine staining was performed. Compared with K562 cells in control group, the K562 cells treated with 5 and 10 *μ*M 6,7-dimethoxycoumarin significantly accelerated the erythroid differentiation, and the K562 cells treated with 1 *μ*M 6,7-dimethoxycoumarin did not make any significant difference (Figures 1(a) and 1(b)). Typical marker of erythroid differentiation such as *α*-globin, *β*-globin, *γ*-globin, CD71, and CD235a was detected by western blot and qPCR. The results showed that compared with control group, the expression of *α*-globin, *β*-globin, *γ*-globin, CD71, and CD235a was significantly decreased in 5 and 10 *μ*M 6,7-Doc group (Figures 1(c) and 1(d)).

### 3.2. 6,7-Dimethoxycoumarin Inhibit the Proliferation of K562 Cell

**Figure 2:**
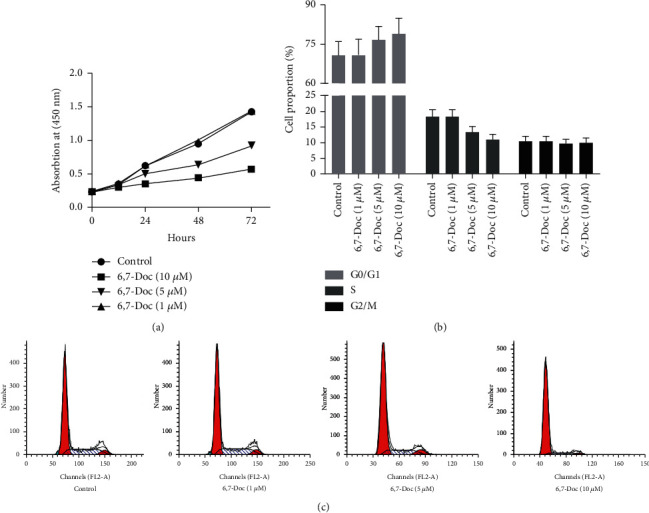
6,7-Dimethoxycoumarin inhibit the proliferation of K562 cell. (a) MTT assay was applied to detect the proliferation of K562 cell. (b, c) The proportion of K562 cells in different cell cycle was evaluated by flow cytometry. ^∗^*P* < 0.05, ^∗∗^*P* < 0.01, compared with control group.

As shown in Figure 2(a), MTT assay was performed to detect the proliferation of K562 cell. The results showed that compared to control group, the proliferation of K562 cells in 5 and 10 *μ*M 6,7-Doc group was significantly decreased, but not significantly different in 1 *μ*M 6,7-Doc group. It suggested that 6,7-dimethoxycoumarin can decrease the proliferation of K562 cell. We further examined the cell cycle of K562 cells (Figures 2(b) and 2(c)). The results showed that 5 and 10 *μ*M 6,7-dimethoxycoumarin induced G0/G1 arrest of K562 cell, which indicated that 6,7-dimethoxycoumarin can inhibited the proliferation of K562 cell.

### 3.3. 6,7-Dimethoxycoumarin Increased the Expression of FOXO3 and Cell Cycle Relevant Proteins

**Figure 3:**
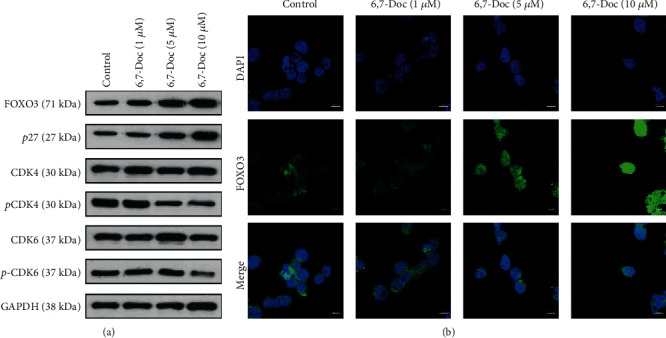
6,7-Dimethoxycoumarin increased the expression of FOXO3 and cell cycle relevant proteins. (a) The expression of proteins was detected by western blot assay. (b) The expression of FOXO3 was detected by immunofluorescence assay. ^∗^*P* < 0.05, ^∗∗^*P* < 0.01, compared with control group.

In order to evaluate how 6,7-dimethoxycoumarin affects the proliferation of K562 cell, we performed western blot and immunofluorescence assay to evaluate the protein expression of FOXO3, P27, CDK4, and CDK6. The results showed that compared to control group, the expression of FOXO3 and P27 was increased in 5 and 10 *μ*M 6,7-Doc group, and the phosphorylation of CDK4 and CDK6 protein was decreased. But the expression of these proteins was not significantly different compared to the control group (Figure 3(a)). The results of immunofluorescence assay showed that compared to the control group, the expression of FOXO3 was significantly decreased in 5 and 10 *μ*M 6,7-Doc group, and not different in 1 *μ*M 6,7-Doc group (Figure 3(b)). The results indicated that 6,7-dimethoxycoumarin increased the expression of FOXO3 and cell cycle relevant proteins.

### 3.4. Knockdown of FOXO3 Notably Reversed the Effect of 6,7-Dimethoxycoumarin on Erythroid Differentiation

**Figure 4:**
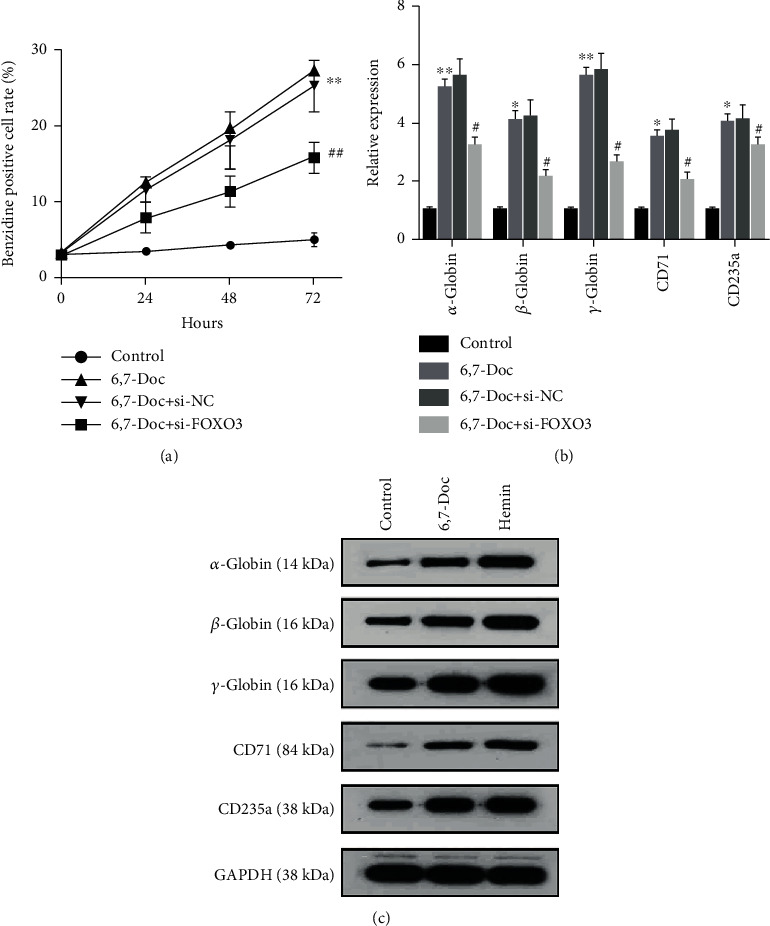
n6-7 Knockdown of FOXO3 notably reversed the effect of 6,7-dimethoxycoumarin on erythroid differentiation. (a, b) Benzidine staining assay was applied to detect the erythroid differentiation. (c) The expression of typical marker of erythroid differentiation was detected by western blot and qPCR assay.^∗^*P* < 0.05, ^∗∗^*P* < 0.01, compared with control group. ^#^*P* < 0.05, ^##^*P* < 0.01, compared with 6,7-Doc group.

From the above results, it has been confirmed that 6,7-dimethoxycoumarin can promote the differentiation and decrease proliferation ability of K562 cell by increasing the expression of FOXO3. In order to further verify the role of FOXO3 in cell differentiation and proliferation, we knocked down the expression of FOXO3 by transfected the plasmid of si-FOXO3 into K562 cells and then verified the differentiation ability of K562 cells by Benzidine staining assay (Figure 4(a)), and further verified the expression of protein such as *α*-globin, *β*-globin, *γ*-globin, CD71, and CD235a by RT-qPCR assay and western blot (Figures 4(b) and 4(c)). The results of the transfection efficiency indicated that transfection with siRNA FOXO3 reduced FOXO3 expression (Figure S1). The results showed that compared with the K562 cells treated with 6,7-dimethoxycoumarin alone, the differentiation ability of K562 cells treated with knockdown of FOXO3 expression and 6,7-dimethoxycoumarin was significantly inhibited, and the expression of cell differentiation relevant protein such as *α*-globin, *β*-globin, *γ*-globin, CD71, and CD235a was significantly decreased. These results indicated that knockdown of FOXO3 notably reversed the effect of 6,7-dimethoxycoumarin on erythroid differentiation.

### 3.5. Knockdown of FOXO3 Notably Reversed the Effect of 6,7-Dimethoxycoumarin on Proliferation of K562 Cell

**Figure 5:**
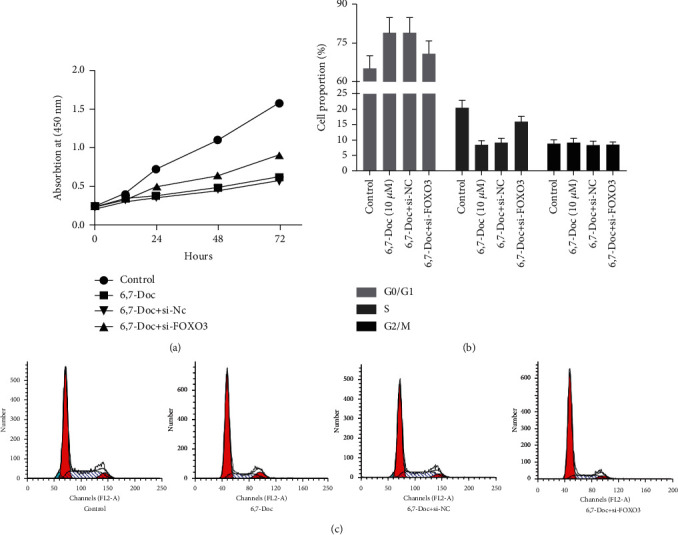
Knockdown of FOXO3 notably reversed the effect of 6,7-dimethoxycoumarin on proliferation of K562 cell. (a) MTT assay was applied to detect the proliferation of K562 cell. (b, c) The proportion of K562 cells in different cell cycle was evaluated by flow cytometry. ^∗^*P* < 0.05, ^∗∗^*P* < 0.01, compared with control group. ^#^*P* < 0.05, ^##^*P* < 0.01, compared with 6,7-Doc group.

Then, we verified the proliferation ability of K562 cell by MTT assay (Figure 5(a)). The results showed that the proliferation ability of K562 cells treated with 6,7-dimethoxycoumarin after knockdown of FOXO3 expression was significantly higher than that of K562 cells treated with 6,7-dimethoxycoumarin alone, and the proportion of cells in different cell cycle was verified by flow cytometry (Figures 5(b) and 5(c)). The results showed that compared with K562 cells treated with 6,7-dimethoxycoumarin alone, the proportion of cells in G0/G1 was significantly decreased in K562 cells treated with 6,7-dimethoxycoumarin after knockdown of FOXO3 expression. To sum up, knockdown of FOXO3 notably reversed the effect of 6,7-dimethoxycoumarin on proliferation of K562 cell.

### 3.6. Knockdown of FOXO3 Notably Reversed the Effect of 6,7-Dimethoxycoumarin on the Expression of FOXO3 and Cell Cycle Relevant Proteins

**Figure 6:**
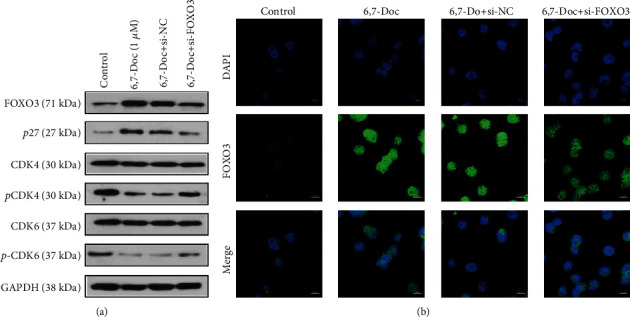
Knockdown of FOXO3 notably reversed the effect of 6,7-dimethoxycoumarin on the expression of FOXO3 and cell cycle relevant proteins. (a) The expression of proteins was detected by western blot assay. (b) The expression of FOXO3 was detected by immunofluorescence assay. ^∗^*P* < 0.05, ^∗∗^*P* < 0.01, compared with control group. ^#^*P* < 0.05, ^##^*P* < 0.01, compared with 6,7-Doc group.

In order to further verify the change in the expression of cell cycle relevant protein after knocking down FOXO3, we carried out western blot assay and immunofluorescence assay (Figures 6(a) and 6(b)). The results showed that compared with K562 cells treated with 6,7-dimethoxycoumarin alone, the expression of P27 was significantly increased, while the expression of phosphorylated CDK4 and CDK6 was significantly decreased in K562 cells treated with 6,7-dimethoxycoumarin after knockdown of FOXO3 expression. These results suggested that knock down of FOXO3 notably reversed the effect of 6,7-dimethoxycoumarin on the expression of FOXO3 and cell cycle relevant proteins.

## 4. Discussion

Chronic myelogenous leukemia (CML) is a fatal malignant tumor. With the continuous development and progress of the medical level, at present, relevant medical technology can improve the survival rate of patients with chronic myeloid leukemia and improve the quality of life. However, it is worth noting that the resulting drug resistance, side effects, and the cost of treatment limit its effectiveness. Therefore, we need to explore new therapeutic targets and discover new drugs, which is undoubtedly a key treatment strategy for chronic myelogenous leukemia.

Recently, with the continuous attention to natural medicines and the in-depth exploration of their active ingredients, natural medicines have certain curative effects, relatively small side effects, and relatively high safety factors. Scopolone is a traditional Chinese medicine phenylpropane monomer. It has a wide range of pharmacological activities, and its antitumor activity has been confirmed in leukemia, laryngeal cancer, and prostate cancer [24, 25]. However, we still need to continue to study and use more convincing evidence to explain the potential molecular mechanism of its antitumor activity.

As a main component of Artemisia capillaris Thunb, 6,7-dimethoxycoumarin has been indicated to play critical roles in cholaneresis and anti-inflammatory, antiasthma, relaxing bronchial smooth muscle. However, it is the first study that focuses on the effect of 6,7-dimethoxycoumarin on erythroid differentiation of human chronic myelogenous leukemia cells.

This study evaluated the antitumor activity of 6,7-dimethoxycoumarin on chronic myeloid leukemia and studied the molecular mechanism of its antitumor effect, providing evidence and theoretical support for potential drugs for the treatment of chronic leukemia. At the same time, it brings certain theoretical support for clinical diagnosis and treatment. Our results revealed that 5 and 10 instead of 1 *μ*M 6,7-dimethoxycoumarin significantly increased the number of erythroid differentiated cells and the expression level of erythroid differentiation biomarkers including *α*-globin, *β*-globin, *γ*-globin, CD71, and CD235a. Moreover, our research also found that 6,7-dimethoxycoumarin also inhibited the proliferation of K562 cell by induce G0/G1 cell cycle arrest.

Hematopoietic cells mainly undergo three processes: cell proliferation, cell differentiation, and cell apoptosis. These processes are closely related to the induction of cell differentiation and the loss of cell proliferation. Cell death is accompanied by the continuous maturation of hematopoietic cells [26]. The cell line K562 is a mature red blood cell differentiation model [27]. Cell cycle arrest is an essential early event in inducing differentiation. The programming of cell differentiation is tightly influenced by cell cycle arrest. Coordinated with cell cycle exit, abundant studies have demonstrated the crucial role of cell cycle arrest during erythroid differentiation. NaBuand derivatives, as HDAC inhibitors, have been shown to induce erythroiddifferentiation of human erythroleukemia cell line K562 through G0/G1 arrest. Sodium butyrate-induced upregulation of p18INK4C gene affects K562 cell G0/G1 arrest and erythroid differentiation.

Chronic myeloid leukemia (CML) is a myeloproliferative disease. It is characterized by chromosomal translocation. The K562 cell line is derived from female embryonic cells with chronic myelogenous leukemia. This cell line is widely used in CML in vitro model system and has also been shown to have an inhibitory effect on cell apoptosis induced by DNA damage [28].

Although we have revealed the effect of 6,7-dimethoxycoumarin on cell cycle arrest and erythroid differentiation of K562 cells, however, the mechanism underlying these function of 6,7-dimethoxycoumarin unclear. We further found that this function of 6,7-dimethoxycoumarin was medicated by FOXO3. Knockdown of FOXO3 using siRNA notably reversed the effect of 6,7-dimethoxycoumarin on erythroid differentiation of K562 cell.

FOXO3 belongs to the FOXO Forkhead family of winged helix transcription factors. In addition to their antioxidant response, FOXO factors exert many fundamental biological functions including the regulation of cell cycle, apoptosis, DNA repair, and metabolism. Among the 4 members of FOXO family, FOXO3 is the major active FOXO during erythroid cell maturation, defective proliferation of Foxo3 mutant erythroblasts that fail to mature. Moreover, FOXO3 was recently demonstrated to be the key for the control of cell cycle progression of immature erythroblasts and their rate of maturation in vivo. The difference is that 6,7-dimethoxycoumarin induced G0/G1 arrest through FOXO3, while in the previous report, it induced G2/M arrest. We further explore the expression of downstream proteins involving in cell cycle. p27 is described as regulating cell cycle progression by inhibiting cyclin-dependent kinases (Cdks) In fact, p27 is also involved in biological functions unrelated to the cell cycle, including the differentiation of red blood cell precursors. Regarding hematopoietic function, p27 is expressed in CD34+ progenitor cells and primitive red blood cell precursors [29]. It is well known that p27, also known as CDKN1B, belongs to the Cip/Kip family and has been identified as potent inhibitor negatively regulates G1-phase cell cycle progression via forming various cyclin–CDK complexes. It is found in complexes composed of Cdks and their cyclin-activating partners and inhibits their catalytic activities. In the present study, we observed a change in the phosphorylation of CDK6 and CDK4. We speculated that p27 helps establish CDK4/CDK6 and cyclin proteins, thus inhibited the activities of CDK4/CDK6.

Disregulation of the cell cycle is a sign of the progress of cancer cells, so preventing the progress of the cell cycle or inducing apoptosis is considered to be an important strategy for cancer treatment [30]. As we all know, cancer cells overcome the surveillance of the host and continue to survive by inhibiting cell apoptosis. Therefore, the exploration and research on the signaling mechanism that promotes the apoptosis of leukemia cells are gradually deepening, in order to guide the production of new therapies for apoptotic genes or pathways.

It is not yet clear whether the anticancer effect of 6,7-dimethoxycoumarin is limited to a small number of cancers. Previous studies on its use in the treatment and improvement of CML are limited. In this study, we evaluated the effect of 6,7-dimethoxycoumarin on the chronic myeloid leukemia cell line (K562).We conduct exploratory research through cell viability, cell cycle, apoptosis, and related gene and protein expression. In conclusion, we determined that 6,7-dimethoxycoumarin obviously promotes erythroid differentiation while inhibit proliferation of K562 cells. Mechanically, it exert these functions through upregulating FOXO3/p27 signal pathway and influence the activities of CDK4/CDK6.

## 5. Conclusion

This study extended the understanding of the pharmacological activity of 6,7-dimethoxycoumarin and may provide a potential new target for the treatment of chronic myelogenous leukemia. However, we still need to further study the specific molecular capabilities of 6.7 dimethylcoumarin to understand their possible capture mechanism.

## Figures and Tables

**Table 1:** Primer sequences.

Gene sequence	Upstream sequence (5′-3′)	Downstream sequence (5′-3′)
CD71	GGACGCGCTAGTGTTCTTCT	CATCTACTTGCCGAGCCAGG
*γ*-globin	CACTCGCTTCTGGAACGTCT	GGTAGACAACCAGGAGCCTTC
*β*-globin	ATCCTGAGAACTTCAGGCTCC	ATTGGACAGCAAGAAAGCGA
CD235a	GCACACTTCAACTTCTTCTTCA	AATAATGAGTGTTATCTCGGTTTCC
*α*-globin	CCGGTCAACTTCAAGCTCCTAA	AAGAAGCATGGCCACCGAGG
GAPDH	AGAAGGCTGGGGCTCATTTG	AGGGGCCATCCACAGTCTTC

## Data Availability

The analyzed data sets generated during the study are available from the corresponding author on reasonable request.

## Supplementary Materials

Supplementary Materials Figure S1: The transfection efficiency of siRNA FOXO3. ∗∗*P* < 0.01, compared with si-NC group.
