# Supplementary material for: 6,7-Dimethoxycoumarin Influences the Erythroid Differentiation of Human Chronic Myelogenous Leukemia K562 Cells through Regulating FOXO3/p27 Signal Pathway
Source: J Oncol. 2022 May 14;2022:1138851. doi: 10.1155/2022/1138851 (PMC9124080; doi:10.1155/2022/1138851)
Supplement: Supplementary Materials — Figure S1: The transfection efficiency of siRNA FOXO3. ∗∗P < 0.01, compared with si-NC group. [file 1138851.f1.docx]

Figure S1. The transfection efficiency of siRNA FOXO3. **P<0.01, compared with si-NC group.
